# Congenic Strain Analysis Reveals Genes That Are Rapidly Evolving Components of a Prezygotic Isolation Mechanism Mediating Incipient Reinforcement

**DOI:** 10.1371/journal.pone.0035898

**Published:** 2012-04-25

**Authors:** Christina M. Laukaitis, Corina Mauss, Robert C. Karn

**Affiliations:** Department of Medicine, College of Medicine, University of Arizona, Tucson, Arizona, United States of America; University of Massachusetts, United States of America

## Abstract

Two decades ago, we developed a congenic strain of *Mus musculus*, called b-congenic, by replacing the androgen-binding protein *Abpa27^a^* allele in the C3H/HeJ genome with the *Abpa27^b^* allele from DBA/2J. We and other researchers used this b-congenic strain and its C3H counterpart, the a-congenic strain, to test the hypothesis that, given the choice between signals from two strains with different *a27* alleles on the same genetic background, test subjects would prefer the homosubspecific one. It was our purpose in undertaking this study to characterize the segment transferred from DBA to the C3H background in producing the b-congenic strain on which a role for ABPA27 in behavior has been predicated. We determined the size of the chromosome 7 segment transferred from DBA and the genes it contains that might influence preference. We found that the “functional" DBA segment is about 1% the size of the mouse haploid genome and contains at least 29 genes expressed in salivary glands, however, only three of these encode proteins identified in the mouse salivary proteome. At least two of the three genes *Abpa27*, *Abpbg26* and *Abpbg27* encoding the subunits of androgen-binding protein ABP dimers evolved under positive selection and the third one may have also. In the sense that they are subunits of the same two functional entities, the ABP dimers, we propose that their evolutionary histories might not be independent of each other.

## Introduction

Strains of mice that differ in only one gene on an otherwise identical genetic background provide a powerful tool for investigating the function of the isolated gene. Twenty years ago, our laboratory developed a mouse strain congenic for a salivary *Androgen-binding protein* (*Abp*) gene in order to test the hypothesis that mouse salivary androgen-binding protein (ABP) is a signal for mouse mate recognition. ABP is a dimeric protein, composed of two subunits originally described as an alpha subunit, encoded by *Abpa*, connected by disulfide bridging to either a beta or a gamma subunit, encoded by *Abpb* and *Abpg*, respectively (these are now a large gene family with a new nomenclature described below). The dimer is capable of binding male sex steroid hormones and progesterone with a relatively high specificity and affinity ([1], reviewed in [2]). Thought at the time to be the only alpha subunit gene in the mouse genome, *Abpa* had been shown to have three different alleles, each fixed in one of the three subspecies of *Mus musculus* (*Abpa^a^* in *M. m. domesticus* (western Europe and the Mediterranean basin), *Abpa^b^* in *M. m. musculus* (eastern Europe to northern China) and *Abpa^c^* in *M. m.castaneus* (Southeast Asia and Malaysia) [3], [4]). This unusual *Abpa* monomorphism, resulting from a different allele having been fixed in each of three different subspecies, suggested that ABP might have a role in mediating subspecies recognition and that idea stimulated our interest in producing strains congenic for different alleles of *Abpa*. The existence of a relatively narrow house mouse hybrid zone between *M. m. domesticus* and *M. m. musculus* in Europe influenced us to choose the alleles from those two subspecies to produce congenic strains.

We crossed DBA/2J (the strain donating the *Abpa^b^* allele) to C3H (the recipient strain; originally C3H/Strong and later C3H/HeJ) which possesses the *Abpa^a^* allele and then backcrossed the F_1_ to the C3H parent strain, selecting the *Abpa^a^*/*Abpa^b^* heterozygote for subsequent backcrosses to C3H. Backcrossing was continued for 16 generations before an intercross was made. The resulting congenic strain was named “b-congenic" and was used in conjunction with the C3H strain (by default “a-congenic") to provide sources of signals in various types of behavioural tests. A locus such as *Abpa*, which has been selected for 16 generations, is expected to retain flanking heterozygosity for an average combined distance of 12 cM (i.e. 12 map units), with a standard deviation of 8 cM [5]. In the mouse genome, a cM is ∼2 Mb of DNA, so the region transferred from DBA to the C3H background to make the b-congenic strain may be as small as 8 Mb or as large as 40 Mb.

Numerous preference tests in Y-mazes conducted in laboratories in the U.S. and Europe, using salivas of C3H and b-congenic strains as sources of signals and either inbred strains and wild-derived, mildly inbred strains [6] or wild-caught individuals [7], [8] as subjects, have confirmed a consistent homosubspecific preference, evidently based on *Abpa* genotype. The most recent of these studies has produced evidence that ABP constitutes an incipient system of reinforcement on the verges of the European house mouse hybrid zone ([8]; this and the foregoing studies are reviewed in [2]).

One important observation from early studies was that subjects do not show a preference for urine from the congenic strains [6], consistent with previous studies showing that ABP is not secreted into urine [9]. Thus the segment transferred from DBA to produce the b-congenic strain apparently does not contain genes for urinary proteins that can influence mate choice. Indeed, the *major urinary protein* (*MUP*) genes, which encode the vast majority of proteins secreted into urine, are on chromosome 4 while the *Abp* genes are on chromosome 7. Because salivas were the sources of the signals used in these tests, we conclude that the product of the gene of interest must be secreted into saliva. This has been previously shown for the three ABP subunits: alpha, beta and gamma [10].

While these behavioral studies were in progress, genomic studies were underway that ultimately revealed that the *Abp* gene region in the mouse is comprised of 3 Mb of DNA on the proximal (with respect to the centromere) end of mouse chromosome 7 and contains 30 alpha (*Abpa*) paralogs, as well as 34 beta-gamma (*Abpbg*) paralogs for a total of 64 *Abp* genes [2], [11]. The chosen *Abpa^b^* allele, now called *Abpa27^b^* [11], has its nearest proximal neighbour (*Abpbg27*, originally *Abpb*) ∼7.6 kb away, while its nearest distal neighbour, *Abpa30* is 76.8 kb away. And what of other genes in the *Abp* cluster? Even though the entire family (3 Mb) comprises 0.1% of the mouse genome, it could nonetheless easily fit into the smallest estimate for the region transferred from DBA. When one considers the larger estimate of the segment transferred (40 Mb), the possibility that other genes on the transferred chromosomal segment might have some effect on the mate choice seen in the behavioral experiments has to be considered.

This project was motivated by two goals: 1) to complement our studies with congenic strains by identifying the genes transferred onto the C3H background that might contribute to the preference seen in mouse behavioural tests; and 2) to investigate the possibility that the signal that stimulates mate choice might be complex, with ABPA27 interacting with another protein(s). We used the mouse genome and genomic sequences of individual strains to determine the size and gene content of the chromosome 7 segment donated by DBA to the b-congenic strain. We combined this information with studies of mouse salivary gland ESTs [12] and proteomes [13] to identify the genes in the transferred chromosome 7 segment that are transcribed by mouse salivary glands and translated into protein secreted into saliva. We examined the genes corresponding to the secreted salivary proteins in the transferred region and found evidence of rapid evolution and positive selection in three of them: *Abpa27*, *Abpbg26*, and *Abpbg27*. This alters our notion of how mouse salivary androgen-binding proteins interact to influence mouse mate preference with at least two, and perhaps three, subunits of ABP dimers comprising the salivary signal.

## Results

### The size of the DBA chromosome 7 segment transferred onto the C3H background in the b-congenic line

**Figure 1:**
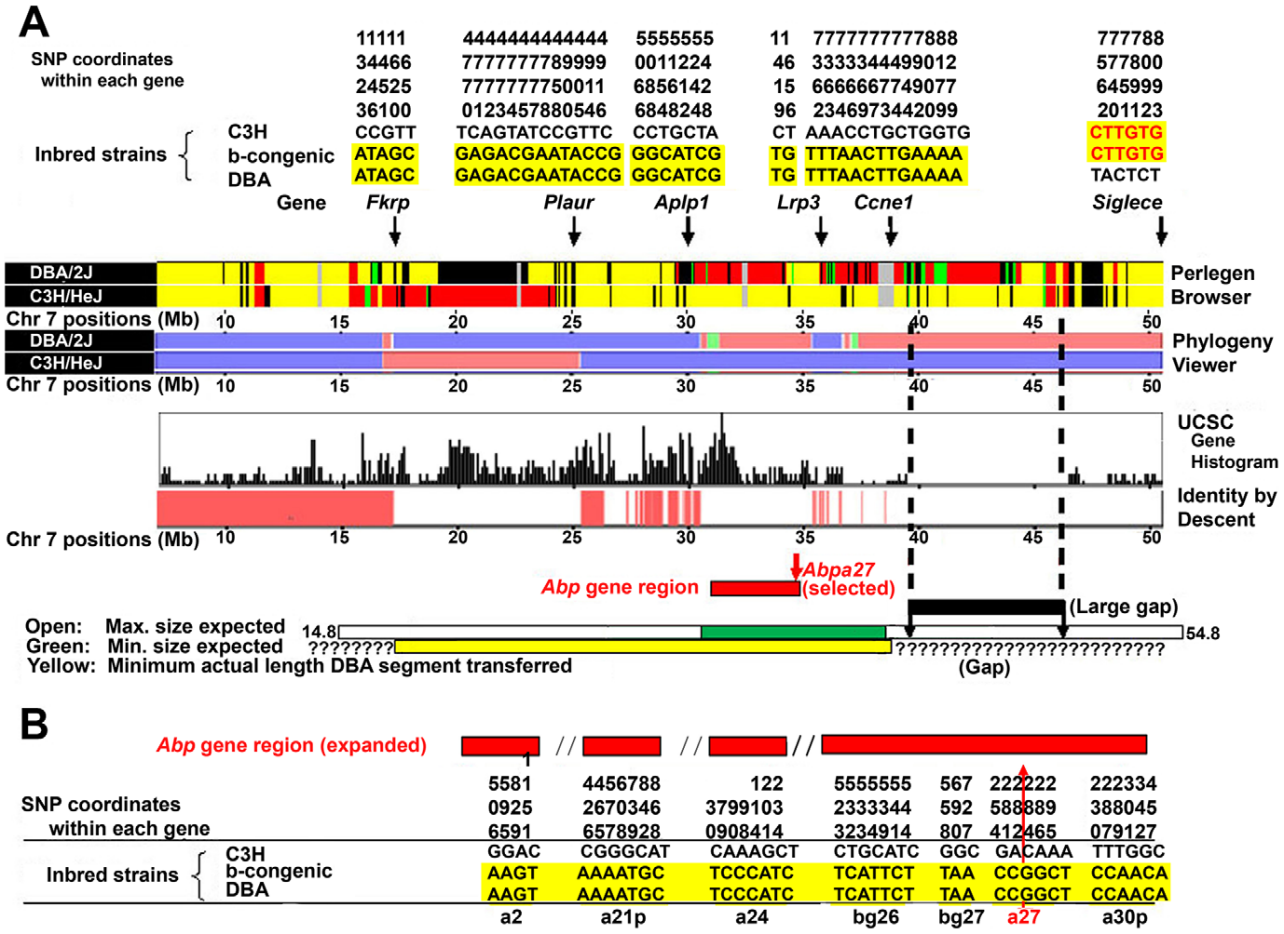
Determining the size and genetic make-up of the chromosome 7 segment transferred from DBA to C3H in producing the b-congenic strain. Panel A: The center of the figure shows the genetic make-up of the chromosome 7 region of interest in the two parent strains, DBA (donor) and C3H (recipient) in terms of the *Mus musculus* subspecies of origin, *Mus musculus domesticus* (yellow in the Perlegen Mouse Ancestry Browser [MAB] and blue in the Mouse Phylogeny Viewer [MPV]); *M. m. musculus* (red in both) and *M. m. castaneus* (green in both). The various regions are shifted slightly in the two because they are based on different builds: mm7 for MAB and mm9 for MPV. The histogram of UCSC genes and the bar representing identity-by-descent (IBD) was obtained from the MPV based on the mm9 genome build. At the top of this panel are the results of typing six genes flanking the *Abp* gene region in the two parent strains and the b-congenic strain. The reduced data sets show only the SNP haplotypes for the two parent strains and their genotypes in the b-congenic strain. Below the MAB and the MPV representations are bars representing the maximum size of the transferred segment calculated from the derived plots of expected combined lengths and standard deviation of heterozygous chromosome segments flanking a selected locus [5] with the maximum size represented as an open bar and the minimum size as a green bar and both centered on the selected locus, *Abpa27*. The proximal end of the transferred segment extends to the left of *Fkrp*, into the shared region that continues nearly to the centromere. The distal end extends no farther than a crossover identified at *Siglece* but might have terminated somewhere in the large gap to the right of *Ccne1*. Panel B: The results of typing three *Abp* genes near the left and right ends of the 3 Mb *Abp* gene region. The reduced data sets show only the SNP haplotypes for the two parent strains and their genotypes in the b-congenic strain. The entire *Abp* gene region appears to have been transferred in the segment donated by DBA to the b-congenic strain.

The Perlegen Mouse Ancestry Browser (MAB) and the Mouse Phylogeny Viewer (MPV) suggest that essentially the entire 3 Mb *Abp* gene region spanning coordinates (cds) ∼32 to ∼35 Mb of DBA chromosome 7 came from *M. m. musculus*, with a small contribution from *M. m. castaneus* (Fig. 1A; mm9/build 37 cds are used here and for other map locations in this paper). Hereinafter *Abpa* and *Abpbg* genes will simply be “*a*" and “*bg*", respectively, each followed by a number in italics. To estimate the size of the DBA chromosome segment transferred onto the C3H background, we first sequenced the genes on the proximal and distal ends of the *Abp* region in the C3H, DBA and b-congenic strains, including *a2*, *a21p*, *a24*, *bg26*, *bg27*, *a27* and *a30p* (see Table S1 for gene information on these and the genes that follow). All these *Abp* genes in the b-congenic strain have the DBA sequence (Fig. 1B).

Three genes on the proximal side of the *Abp* region, *Fkrp*, *Plaur* and *Aplp1* were sequenced in the b-congenic strain and found to have DBA SNPs (Fig. 1A). The >10 Mb region from the left of *Fkrp* extending leftward nearly to the centromere appears to be derived from *M. m. domesticus* and is identical by descent (IBD) in the two strains. Thus it is not possible to determine exactly the left boundary of the transferred portion. Two genes on the distal side of the *Abp* region, *Lrp3* and *Ccne1* have the DBA SNPs in b-congenic, while the more distal gene *Siglece* has C3H SNPs in b-congenic. This suggests that a crossover occurred between cds ∼38.8 and ∼50.9 during backcrossing. There is a large gap between cds ∼39.5 and ∼46 Mb that begins almost immediately to the right of *Ccne1* (see Table S2 for cds of gaps in this region of chromosome 7). This gap limits the possibility for making a better estimate of where the transferred chromosomal segment ended distal to the *Abp* region. Nonetheless, the cross-over proximal to *Siglece* defines the furthest possible distal reach of the chromosome segment transferred from DBA.

Regardless of its actual size, the “functional" size of the segment transferred from DBA to the b-congenic strain, i.e., the region where the two parent strains differ in the subspecies source of their segments, is ∼34 Mb. This is calculated as the distance from the first point beyond the chromosome 7 centromere (cd ∼17.3), where the two parental strains differ in their subspecies genetic contribution, to the crossover somewhere proximal to *Siglece* at cd ∼50.9. While it is not possible to determine the exact left terminus of the transferred segment, this is irrelevant for our study because it was derived from *M. m. domesticus* in both C3H and DBA and thus their genes will have the same subspecies origin in the region IBD. These considerations lead to the interesting conclusion that the segment is nearly as large as the 40 Mb maximal theoretical estimate (Fig. 1). Gene(s) encoding the signal recognized by female mouse subjects must lie in this 34 Mb region.

### Candidate genes

We used two different analyses to search for candidate genes: 1. Searching ESTs expressed in mouse salivary glands [12] for those encoded by genes located on the portion of chromosome 7 that might have been transferred onto the C3H background. 2. Searching recently published mouse saliva proteomes [13] for evidence of proteins expressed by genes in the chromosome 7 region of interest and secreted into saliva. This analysis allows us to determine which of the apparent expressions from analysis 1 actually produce proteins identified in mouse saliva.

**Figure 2:**
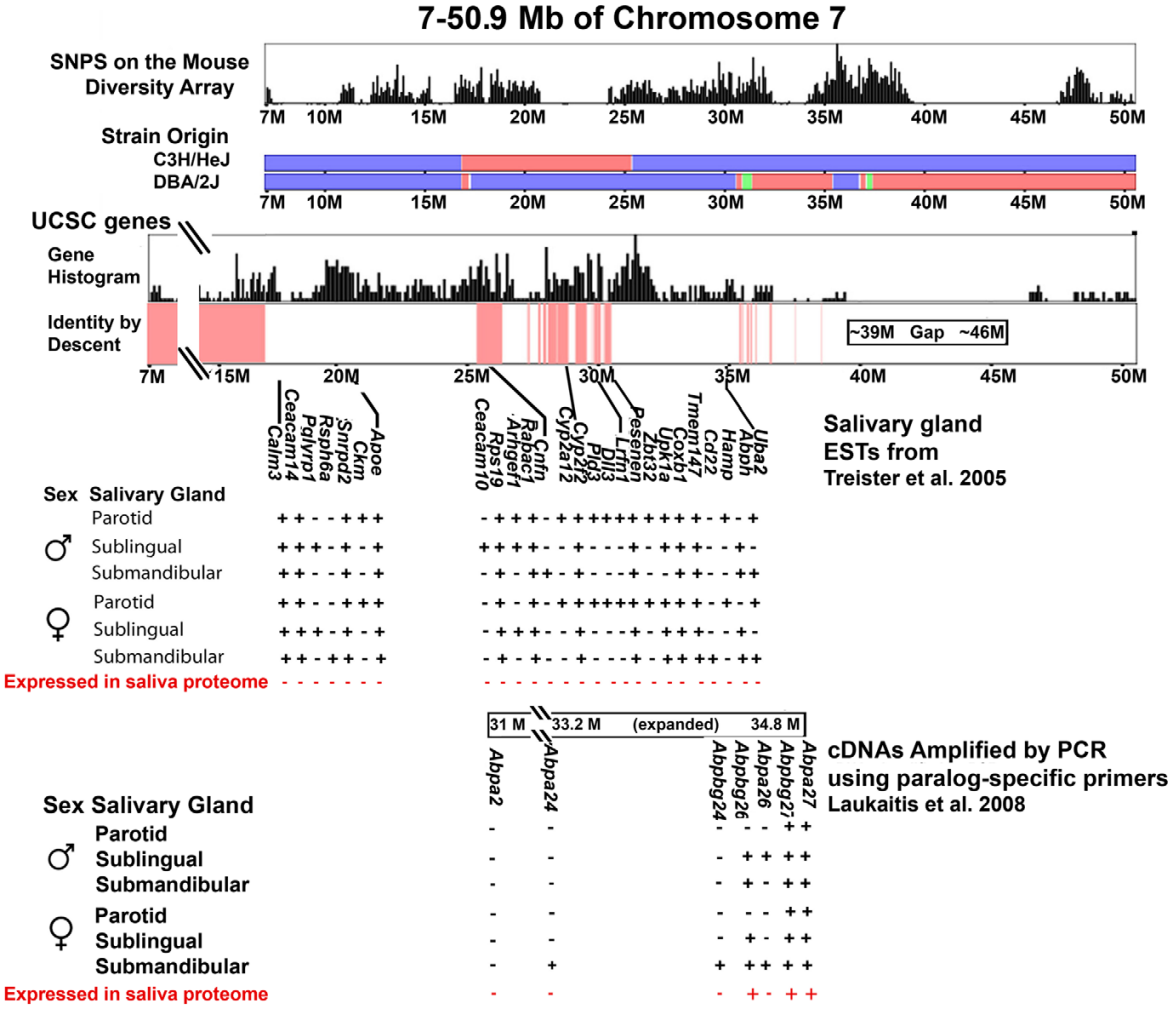
Locations of salivary gland ESTs on the chromosome 7 region of interest (cds 7–50.6 Mb). For reference, the subspecies origin of both parent strains as determined from the Mouse Phylogeny Viewer (MPV) is shown on the top bars (*Mus musculus domesticus* in blue; *M. m. musculus* in red and *M. m. castaneus* in green) with the density of SNPs above them. The UCSC gene histogram and bar representing identity-by-descent (IBD) is shown below the bars and the salivary gland ESTs from two different studies are arrayed in the bottom two tiers of the figure. The salivary gland EST data of [12] was sorted to obtain only the ESTs encoded on the chromosome 7 region of interest. *Abp* cDNAs amplified by PCR using paralog-specific primers [14] are arrayed at the bottom of the figure.

These two analyses are complementary and allowed us to make the most thorough search possible with the data currently available. For the first analysis, we downloaded the ESTs reported from a study of gene expressions in all three major salivary glands, parotid, sublingual and submandibular, evaluated in both sexes in the BALB/c strain [12]. We sorted the BALB/c data subsets on chromosome 7 to retain those with genes located between cd ∼17.3 Mb and 50.9 Mb where C3H and DBA differ in the subspecies origins of their genomes (Fig. 2; Table S3). Other studies have reported ESTs from only one gland and just one sex in other inbred strains, however, mouse submandibular glands show sex-limited developmental differences at puberty. Male mice elaborate granulated convoluted tubular tissue [13] causing submandibular gland enlargement and expression of salivary proteins not found in female salivas, including 12 members of the mouse-specific subfamily b kallikreins [13].

There were 26 genes within the segment described above (Fig. 2). Ten of these (38%) were expressed in all three salivary glands of the mouse, while seven (27%) were restricted to expression in the parotid, one to the sublingual (4%) and none were found only in the submandibular. These 26 genes were examined to determine which ones encode proteins with strictly intracellular (e.g. ribosomal proteins) and/or membrane-bound (e.g. Rab acceptor 1) localization, and which ones encode exocrine proteins potentially secreted into saliva (File S1). The sequences of some coding regions which might produce secreted proteins were compared between C3H and DBA in the Sanger Mouse Genomes Project data (File S2). Genes were eliminated if they had either no nonysynonymous substitutions or only one or two that made conservative amino acid substitutions (File S1).

Data from male and female mouse saliva proteomes [13] were compared with EST data to determine which genes in this region of chromosome 7 were candidates for the signal influencing female choice in preference tests (Fig. 2; Table S4). Reducing identification stringency [13] increased the number of salivary protein identifications from 81 to 730. In spite of that, we did not find evidence that any of the 26 ESTs gleaned from our analysis correspond to proteins identified in the mouse saliva proteomes. For most of the ESTs, this result is not surprising because they have been identified as intracellular and/or membrane-bound in their localizations and do not have characteristics of exocrine proteins (File S1). By contrast, the *bg26*, *bg27* and *a27 Abp* paralogs that had salivary gland cDNAs identified by [14] were some of the most highly represented proteins in the Scaffold array under the most stringent criteria [13]. The only other genes encoded on chromosome 7 expressed in the mouse saliva proteome were the genes for nucleobindin and the salivary kallikreins (*Klk1* and *Klk1b5* in both sexes, and 12 other *Klk1b* family genes in males only). All these genes map distally to *Siglece* and thus outside the region transferred from DBA to the b-congenic strain.

### Evolution of candidate signal genes

**Figure 3:**
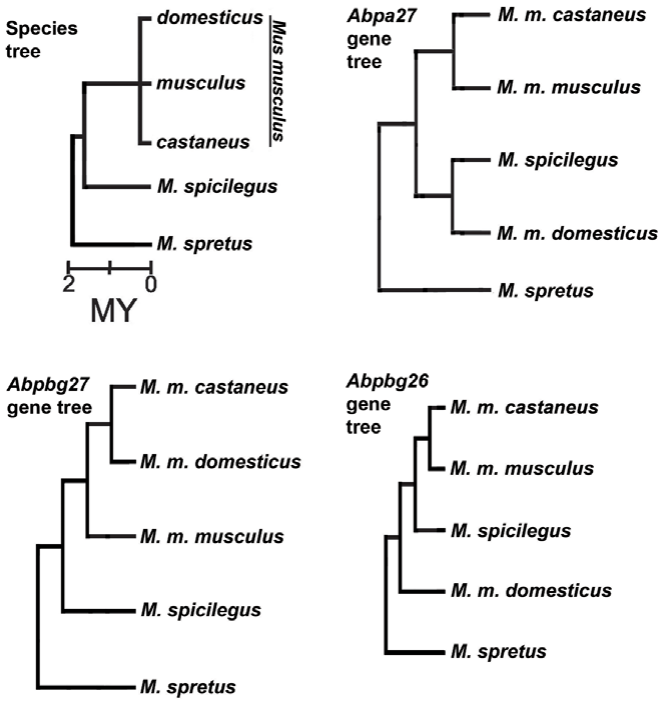
Gene trees for *Abpa27*, *Abpbg26* and *Abpbg27* compared to the species tree of the genus *Mus*. Taxa used in this study include the three subspecies of *Mus musculus* (*M. m. domesticus*, *M. m. musculus* and *M. m. castaneus*), *M. spicilegus* and *M. spretus*. The species tree is modified from [17] with the three subspecies of *Mus musculus* represented as an unresolved polytomy. The gene trees for *Abpa27*, *Abpbg26* and *Abpbg27* were made by aligning the sequences of each of the five taxa in Clustal X and using the alignments to make neighbor-joining trees in PAUP* (see methods for details). Each of the four trees was rooted on the *M. spretus* sequence. Only the gene tree for *Abpbg27* is congruent with the species tree. The gene trees for *Abpa27* and *Abpbg26* both show the *M. m. domesticus* sequence out of place relative to those of the other taxa.

The three closely linked *Abp* genes, *a27*, *bg27* and *bg26* map in the chromosome segment transferred from DBA and encode proteins found in saliva. C3H and DBA cDNA and protein sequences of these genes were published previously [15], [16]. *bg26* has the most nonsynonymous substitutions (40 causing 32 amino acid substitutions; [16]), *a27* has five, causing four amino acid substitutions [15] and *bg27* has the least (three, each causing an amino acid substitution; [16]). We obtained the sequences of the *a27*, *bg27* and *bg26* genes in five taxa (File S3), including the three subspecies of *Mus musculus*, as well as *Mus spicilegus* and *Mus spretus* and constructed gene phylogenies. The *bg27* gene phylogeny was congruent with the species tree but the gene phylogenies for *a27* and *bg26* were not (Fig. 3; File S4).

The phylogeny of [17] was used for the rodent species guide tree for the CODEML tests and the three subspecies of *M. musculus* were treated as an unresolved polytomy (Fig. 3; [18], [19]). Two of the three genes, *a27* and *bg26* showed significant signs of positive selection within these five murid rodents when the species phylogeny was used as the CODEML guide tree (Table 1). On the other hand, gene phylogenies for *a27* and *bg26* suggest that the evolution of these two genes may not be accurately represented by the species tree, but are better supported by an alternate tree (Fig. 3, File S1). While this had been observed previously for *a27* [4], [15], this is the first report of gene and species trees for the two *bg* subunits. Differences in gene and species trees may be explained by phenomena including one or more introgression events, incomplete lineage sorting, and/or homoplasies (recurrent mutations). Such introgression or homoplastic mutations might even have been fixed due to positive selection, and the gene trees for *a27* and *bg26* are curiously similar insofar as it is the *M. m. domesticus* branch that is out of place in both. While some investigators feel that the evolutionary relationships among the three full Palearctic species of *Mus* may be tenuous (e.g. [20]–[22]), PAML's author has suggested that the gene tree should be used when it differs substantially from the species tree (http://www.ucl.ac.uk/discussions/viewtopic.php?t  =  7850). Thus the gene tree was the appropriate choice regardless of the status of the species tree.

**Table 1:** Results of selection test on *Abpa27*, *Abpbg26* and *Abpbg27*.

	Using the Species Phylogeny			Using the Gene Phylogeny		
Gene	Ratio of *dN/dS* (%Codons)^a^	*P* Value All Species^b^	Codon Sites Under Selection^c^	Ratio of *dN/dS* (%Codons)^a^	*P* Value All Species^b^	Codon Sites Under Selection^c^
*Abpa27*	16.8 (12.7%)	<0.0002	**32T**, **33K**, **36E**, **39A**	11.9 (14.6%)	0.01	**32T**, **33K**, 36E, **39A,**
*Abpbg26*	3.19 (25%)	0.004	**5A**, 7T, 11I, **14L**, **15R**, 21G, **27Y**, **39I**, **44R**, **48Q**, 75L	12 (4.6%)	0.007	**15R**, **48Q**
*Abpbg27*	6.1 (37%)	0.14	**9V**, **59M**, **72P**	The gene and species phylogenies are identical for bg27		

a The *dN/dS* ratio of the class of codons under positive selection is given with the percentage of codon sites predicted to be in that class.

b The p-value rejecting the model of neutral evolution (M8A) over that of selection (M8) is given.

c Sites with posterior probabilities greater than 0.9 are indicated in regular typeface; p>0.95 indicated in bold typeface and p>0.99 indicated in bold, underlined typeface.

When *a27* and *bg26* were analyzed with CODEML using their gene phylogenies as the guide trees, we again obtained significant signs of positive selection within these five murid rodents (*a27* P = 0.01 with an estimated 14.6% of codons showing a *dN/dS* ratio of 11.9; *bg26* P = 0.007 with an estimated 4.6% of codons showing a *dN/dS* ratio of 12; Table 1). It seems likely that the proteins encoded by both *a27* and *bg26* evolved under positive selection but, by contrast, the gene tree for *bg27* was congruent with the species tree (Fig. 3) and the CODEML result was non-significant (P = 0.14 with an estimated 37% of codons showing a *dN/dS* ratio of 6.1).

Because of the evidence for positive selection on ABPA27 and ABPBG26, we predicted positively selected amino acid sites using a Bayes empirical Bayes (BEB) method ([23]; Table 1). While selected sites identified by analysis of paralogs as a group have been reported [24], this is the first time that *Abp* orthologs from various taxa have been analyzed to identify specific sites under selection in each. Four amino acid sites in ABPA27 were identified as positively selected at a BEB posterior probability threshold of 90%, regardless of which tree was used. In the case of ABPBG26, however, eleven sites were identified as positively selected at a BEB posterior probability threshold of 90% when the species tree was used as the guide tree but only two when the gene tree was used. Thus, the gene tree produced a more conservative result. In spite of the non-significant CODEML result for ABPBG27, three amino acid sites were identified as positively selected at a BEB posterior probability threshold of 95%. Whether or not to report positively selected amino acid sites obtained with the BEB method when the CODEML result is non-significant is controversial (W. Swanson, personal communication). While the outcome (M8 - M8a) was not statistically significant, the current test is underpowered [25]. However, the BEB sites indicated with a high posterior probability of being under positive selection are still of interest and represent interesting targets for future functional analysis and so we report both results here with that caveat.

It is possible that adding taxa to the CODEML analysis would have tipped the scales from nonsignificant to significant for the *bg27* gene. We attempted to amplify segments from *Mus caroli* (an Asian species; the others are Palearctic species) genomic DNA using the *bg26* and *bg27* primer sets, and sequenced a candidate from the *bg26* amplification. Attempts to verify orthology of the *bg26* candidate using BLAT suggested that it was more closely related to *bg27*. While that seemed to qualify this as a sixth sequence for the purpose of re-running the *bg27* CODEML analysis, the *M. caroli* sequence is radically different from any of the original five. More problematic is the observation in *M. caroli* of two, closely related *a27* paralogs in the clade containing *a26p*, *a27* and *a30p* [11]. Thus the orthology of the four genes in the <*bg26-a26p*> and <*bg27-a27*> modules in *M. caroli* with their counterparts in the other species and subspecies of *Mus* is obscured by the possibility that their evolutionary history is different in the *M. caroli* genome than in those of Palearctic species used in this study.

**Figure 4:**
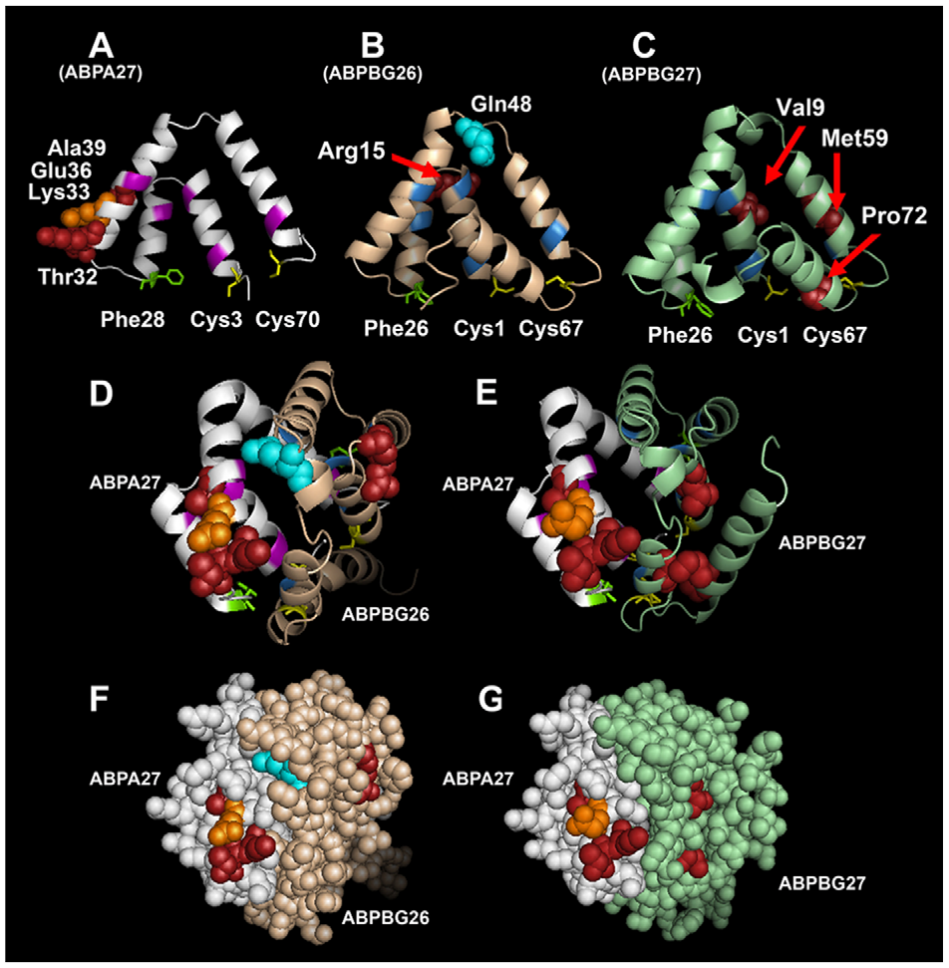
Positive selection at sites on molecular models of mouse salivary ABPA27 and ABPBG26 and the dimer they form. Panels A–E are cartoon-format models with spiral ribbons representing alpha helices and thinner connecting lines representing loops. Gene trees were used for the CODEML analyses of the coding regions of *bg26* and *a27*, the species tree for *bg27*. Panels F and G are illustration of the dimers composed of the two subunits with amino acid residues as filled spheres. Panels A–C show inside views of the three ABP subunits: ABPA27 (white), ABPBG26 (tan) and ABPBG27 (light green), respectively. The conserved residues that line the ligand-binding pocket are represented in purple for the ABPA27 model and blue for the ABPBG26 and ABPBG27 models, with the conserved Phe that coordinates the orientation of the monomers shown in green with stick side chain. The Cys residues that form disulfide bridges uniting the monomers in antiparallel orientation are shown in yellow with stick side chains. The residue in bright blue is selected with a BEB posterior probability of 99%; those in red with BEB posterior probabilities of 95%; and the one in orange with a BEB posterior probability of 90%. Panel D: the ABPA27-ABPBG26 dimer in cartoon format. Panel E: the ABPA27-ABPBG27 dimer in cartoon format. Panel F: a solid model of the ABPA27-ABPBG26 dimer showing all six selected residues on one face. Panel G: a solid model of the ABPA27-ABPBG27 dimer showing six of the seven selected residues on one face (one of the ABPBG27 selected residues is out of sight on the left side).

Using the Phyre2 threading program, we predicted three-dimensional structures for the ABPA27, ABPBG26 and ABPBG27 monomers, as well as the A27-BG26 and A27-BG27 dimers they form (Table 2). We plotted the sites selected at a BEB posterior probability threshold of 90% on all three models with Pymol (Fig. 4), using the sites from the more conservative gene tree analyses for *a27* and *bg26*, and the species tree analysis for *bg27* (Table 1). All the selected sites in the three monomers fall on their exteriors. The solid-filled models of the two dimers show that all six selected sites fall on one face of the A27-BG26 dimer, while six of the seven selected sites fall on the same face of the A27-BG27 dimer.

**Table 2:** Mouse genes used to produce molecular models.

Monomers/Dimers	Accession Number	Chromosomal Location (strand)	Structural Model^a^ (% confidence; length; and % identity)
*Abpa27*	GU269231	chr7:34,806,587–34,807,768 (+)	c1puoA (100%; 70; 51%)
*Abpbg26*	AY293279	chr7:34,796,951–34,798,961 (−)	c1puoA (100%; 84; 28%)
*Abpbg27*	AY293282	chr7:34,728,016–34,730,004 (−)	c1puoA (100%; 84; 28%)
*Abpa27*- *Abpbg26*	N/A - constructed from monomers		c2ejnB (100%; 136; 43%)
*Abpa27*- *Abpbg27*	N/A - constructed from monomers		c2ejnB (100%; 135; 42%)

a The secreted sequences (i.e., signal sequences removed) were threaded for this study.

## Discussion

The b-congenic strain and its counterpart, the C3H parent strain (a-congenic) served as the foundation of a number of studies aimed at testing the hypothesis that, given the choice between signals from two strains with different *a27* alleles on the same genetic background, test subject mice would show a preference for the homosubspecific one [26]. The most commonly used study design involved two-way choice testing in a Y-maze and has confirmed the original hypothesis in laboratory mice [6] and wild mice [7]. Most recently provided evidence that ABP forms the basis of incipient reinforcement on the hybrid zone where two subspecies of *M. musculus* made secondary contact [8].

### Why analyze a congenic strain?

We undertook the project reported here to determine the physical and genetic nature of the chromosome 7 segment transferred from DBA onto the C3H genome to produce the b-congenic strain. We employed the information in the mouse genome [27], an EST database [12] and mouse saliva proteomes [13] We wished to learn whether the results of the many behavioral tests that relied on the b-congenic strain were the direct affect of the *a27^b^* allele, as originally intended. The alternative hypothesis is that they are attributable to the affect of another, closely linked gene that is also expressed in saliva and that differs significantly in the two parental strains. Another possibility that must be considered is that one or more *Abp* genes (*a27* and/or another *Abp* gene) is responsible for the preference test results but those results were also influenced by the expression of an additional, non-*Abp* gene that also encodes a protein secreted into saliva and that differed significantly in the parental strains.

At the time that we began breeding the b-congenic strain, only three *Abp* genes had been predicted from biochemical and genetic studies [10]. This affected the initial behavioral studies that used the b-congenic strain in the mid-1990s because the assumption behind the experimental design [26] was that the gene, then called *Abpa*, that encoded the alpha subunit of ABP was the only one encoding that subunit common to two dimeric forms found in mouse salivas: alpha-beta and alpha-gamma (beta encoded by *Abpb* and gamma by *Abpg*; [10]). The original three-gene hypothesis was eventually confirmed [28], however, as that study was in progress in 2002, the draft mouse genome sequence was being completed [27] and it became apparent that there were many more *Abp* genes than just those three. A collaboration between the laboratories of Karn in the U.S. and Ponting in the U.K. produced a number of papers (e.g., [14], [24]) culminating in the description of 64 *Abp* paralogs covering a span of 3 Mb in the completed *Abp* region on chromosome 7 [11]. Although this region encompasses ∼0.1% of the mouse genome, it is still small enough to have been included in the lower estimate (24 Mb) of the region transferred from DBA onto the C3H genome in producing the b-congenic strain. Thus it is possible that one or more of the other *Abp* genes lying close to the selected gene, *a27*, contribute to the preference of subjects in the Y-maze tests. This increased the complexity of the question regarding just what genes are congenic in the b-congenic strain we produced.

### The size and gene content of the segment contributed by DBA to the b-congenic line

In spite of a large region of *M. m. domesticus* contribution to both strains' chromosome 7 and a number of gaps, we were able to narrow down the “functional" size of the segment to ∼34 Mb. This is not appreciably smaller than the upper value of 40 Mb estimated from the exact derivation of [5]. The mm9 cds of our estimate extend from ∼17.3 Mb to ∼50.9 Mb, delineated by the common region in the parental strains proximal to 17.3 Mb and by the region on the right, distal to the large gap (cds ∼39 to ∼46 Mb) where there is a crossover in the *Siglece* gene. This determination of the size of the segment is important because it delimits its gene content. For example, it excludes the vomeronasal receptor genes proximal to cds 17.3 Mb and the kallikrein genes distal to cds 50.9 Mb. More importantly, it allows us to look for genes included within its span that are transcribed in one or more of the three salivary glands and secreted into saliva. This region of chromosome 7 contains many genes, most of which are not transcribed in any of the three salivary glands. We searched the results of a study that identified salivary gland ESTs [12] and found 26 that map to the segment transferred from DBA chromosome 7. The one characteristic that all of the 26 proteins in this group shared was that we could find no evidence in the data from a recent mouse proteome for any of their peptides in either male or female mouse saliva, even when the criteria in the Scaffold software analysis were relaxed substantially [13]. This is not entirely unexpected given that the segment in question is little more than ∼1% of the mouse genome. Moreover, we could expect that many of the ESTs in the three salivary glands will represent the expression of genes encoding proteins such as those required for cell function and those produced by other types of cells that do not secrete exocrine proteins.

One protein, *Abph* (aka *Abpa2*) was listed as showing a medium level of expression in microarray analysis of the sublingual and submandibular glands, however, there was no evidence of a transcript for this *Abp* gene in: 1) extensive testing of parotid gland, sublingual gland and submaxillary gland [14], 2) cDNA libraries from C3H and DBA strains [16], 3) saliva from androgen-binding and immunostaining studies [10], or 4) a recent saliva proteomics analysis [13]. We conclude that the *Abph* (*Abpa2*) expression in sublingual and submandibular glands [12] was probably a misidentification caused by the binding of transcripts from other, highly expressed *Abp* genes (see below) to the probes produced from the *Abph* transcript for the study. Of the 64 *Abp* paralogs in the mouse genome there is only evidence that three of them, *bg26*, *bg27* and *a27*, are transcribed in the three salivary glands [14] and their gene products are ecreted into saliva in significant amounts [10], [13]. There is no evidence for the expression of any of the 61 other *Abp* paralogs.

### The genes encoding the preference signal and their evolutionary characteristics

It appears, in light of the proteome data [13], that *a27*, *bg26* and *bg27* are the only genes identified on the segment of chromosome 7 transferred to the b-congenic strain that could have influenced the results of the Y-maze preference tests [6]–[8]. While this study makes it unlikely that other genes may have been involved, we cannot absolutely rule that out. Each of these three genes has a substantial number of nonsynonymous substitutions in its coding region. We investigated the evolutionary history of both of these *Abpbg* genes by comparing their gene phylogenies and by using the CODEML program to look for the footprints of adaptive evolution.

Our CODEML analysis supports the notion that both subunits, A27 and BG26, have a history of adaptive evolution driven by positive selection on a few sites at the surface of one face of the dimer they form. We suggest that these two genes evolved rapidly as subunits that form one of the dimers secreted in relatively large quantity into the salivas of mice and that their evolutionary histories may not be independent. This is not unexpected given that at least A27 appears to be involved in incipient reinforcement on the hybrid zone where *M. m. domesticus* and *M. m. musculus* made secondary contact [8] but it must do so in conjunction with at least one BG monomer because all ABP subunits described to date are paired in an alpha-beta/gamma dimer.

Four amino acid sites of A27 apparently evolved under positive selection as have at least two of BG26. Whether the rapid evolution of A27 and BG26 has been shared by the BG27 subunit is less clear. The CODEML result for *bg27* was non-significant, however, there were three sites in the sequence of BG27 identified as positively selected at a BEB posterior probability threshold of >95%. As noted above, whether to accept such sites as selected, given the non-significant CODEML result, is controversial but it is nonetheless interesting that two of the three sites are at the surface of one face of the dimer formed with the A27 subunit resulting in a model that looks very similar to that of the A27-BG26 dimer (Fig. 4).

The CODEML results for *a27*, *bg26*, and *bg27* suggest that at least A27 and BG26 may have coevolved. These are the subunits of the same functional entity, the A27-BG26 dimer found in mouse saliva. BG27 may have also coevolved with A27 because these two subunits form a dimer found in mouse saliva at a level nearly equal-molar with the A27-BG26 dimer [29]. Both of the residues under selection in BG26, and two of the three residues under selection in BG27, share the same exterior face of the dimer with the four residues under selection in A27. This location of all but one of these selected residues suggests that these ABP dimers interact with another molecule(s). It is interesting to compare the results we report here with those of [29] who showed that the two different dimers, A27-BG26 and A27-BG27 bind dihydrotestosterone (DHT) and testosterone (T), respectively, with different affinities. Although it is not clear how amino acid variation at sites on an exterior face of the molecule might make this possible, it is likely that the different binding affinities result from a synergistic affect caused by the interaction of A27 with the BG monomer in each of the two dimers. One possibility is that conformation of the binding pocket depends on which BG subunit is involved, since this pocket is created by the formation of the dimer [30]. Indeed BG26 and BG27 differ at more amino acid sites than the ones shown here to be evolving under positive selection [16]. Thus, while coevolution of A27 with one or both BG26 and BG27 can affect a surface interaction with another molecule, changes at other amino acid sites independently, or in conjunction with the positively selected site, can affect ligand binding.

We produced the b-congenic strain to test the hypothesis that, given the choice between signals from two strains with different *a27* alleles on the same genetic background, the test subject mice would show a preference for the homosubspecific one [26]. At the time that project was undertaken, it was clear that whatever affect A27 has on behavior, it does so as a subunit of a dimer with either BG26 or BG27, since no free monomers have been observed in mouse salivas [10]. Thus it is not surprising that these genes, closely linked to *a27* and sharing its evolutionary history [11], should both also have a role in mediating mate preference behavior. It seems evident that we can conclude this about the A27-BG26 dimer at the very least and perhaps about the A27-BG27 dimer as well.

Our analysis of the segment transferred from DBA in the process of breeding the b-congenic strain reinforces and extends the role of ABP in the many studies done with saliva from the congenic strains. The most exciting finding, however, is that the gene used for selection in the congenic breeding, *Abpa27*, encodes only one component of the salivary proteins that influence assortative mate choice. We have presented evidence here that at least one, and perhaps both of the genes, *Abpbg26* and *Abpbg27*, encoding the other members of the two salivary ABP dimers shows the characteristics of adaptive evolution. We suggest that the two different ABP dimers in saliva exist to modulate the stimulus, especially if their different affinities for the steroid ligands T and DHT also influence mate choice.

## Materials and Methods

All animal manipulation was performed humanely and under appropriate Animal Welfare Guidelines under University of Arizona IACUC protocol 08-138. Methods are summarized here; details appear in File S5.

Polymerase chain reaction (PCR) was run as previously described [14] using genomic DNAs either purchased (Jackson Laboratory) or purified from tail tips and the products sequenced either by the UAGC facility at the University of Arizona or by MCLAB (http://www.mclab.com/). DNA sequence traces were edited with Chromas 2.3 (http://www.technelysium.com.au). DNA sequence alignment, coding region assembly, and *in silico* translation were done using the DNAsis Max program 2.0 (Hitachi).

Salivary gland protein expression data sets were obtained by searching “mouse salivary gland" on the NCBI Gene Expression Omnibus website (www.ncbi.nlm.nih.gov/geo/). Data was downloaded and cross-referenced with Amersham Codelink UniSet Mouse 1 Bioarray, and protein information for each probe target was gathered by searching probe accession numbers on MGI (http://www.informatics.jax.org/), the international database resource for the laboratory mouse. The data in the region of interest was also sorted on the sample signal intensity from the array, which ranged from 0 to 250–400 in the six data sets, and only those with values of 10 or above were retained for this study.

Positive selection was assessed in the program CODEML in the PAML package [31]–[33]. The three-dimensional structures of mouse *a27*, *bg26* and *bg27* were modeled using the PHYRE2 (version 2.0) threading program (http://www.sbg.bio.ic.ac.uk/phyre2/html/page.cgi?id=index; [34]), and the resulting models were visualized using PYMOL (open-source 1.2.8; http://www.pymol.org/). Sites under positive selection were mapped onto the structural models using PYMOL and incorporated into a figure.

## Supporting Information

Table S1 **Gene information.**
(XLS)

Table S2 **Sequence gaps (regions with low/no SNP density in the Mouse Diversity array and lacking UCSC genes).**
(XLS)

Table S3 **ESTs found in one or more of three glands of the two sexes of BALB/c mice** [**12**] **.**
(DOC)

Table S4 **Chromosome 7 encoded proteins found in mouse saliva proteomes** [**13**] **.**
(XLS)

File S1 **Retained and eliminated proteins (represented by ESTs and cDNAs).**
(DOC)

File S2 **Protein sequences from Sanger Data and from sequencing.**
(TXT)

File S3 **Sequences of Abp coding regions used in CODEML analysis.**
(TXT)

File S4 **Unrooted trees for** ***Abpa27*** **,** ***Abpbg26*** **and** ***Abpbg27*** **.** Trees were made as in Fig. 3 but were not rooted. Rather, the suggested root is shown by a black dot on the branch to the *Mus spretus* ortholog.
(TIF)

File S5 **Details of Materials and Methods.**
(DOC)
