# Supplementary material for: Congenic Strain Analysis Reveals Genes That Are Rapidly Evolving Components of a Prezygotic Isolation Mechanism Mediating Incipient Reinforcement
Source: PLoS One. 2012 Apr 25;7(4):e35898. doi: 10.1371/journal.pone.0035898 (PMC3338474; doi:10.1371/journal.pone.0035898)
Supplement: Table S3 — ESTs found in one or more of three glands of the two sexes of BALB/c mice [12] . (DOC) [file pone.0035898.s003.doc]

Table S3. ESTs found in one or more of three glands of the two sexes of BALB/c mice

Protein in

Chromosome 7 saliva Additional

Gene Description Region proteome^1^ observations

*Calm3* Calmodulin 17,500,728-17,509,463 No Intracellular protein

*Ceacam14* Carcinoembryonic antigen-related

cell adhesion molecule B1 18,397,982-18,401,012 No Tumor marker for carcinomas

*Pglyrp1* Peptidoglycan recognition protein 1 19,470,039-19,475,787 No Intracellular splicing protein

*Rsph6a* Radial spoke head 6 homolog

(Chlamydomonas) 19,640,039-19,659,796 No Belongs to the flagellar radial

spoke RSP4/6 family

*Snrpd2* Small nuclear ribonucleoprotein D2 19,735,071-19,738,084 No Intracellular protein

*Ckm* Creatinine kinase, muscle 19,996,443-20,006,932 No Large gap in one strain

*Apoe* Apolipoprotein E 20,281,595-20,284,515 No Glu163 to Asp163

*Ceacam10* Carcino-embryonic antigen-related Region identical by descent

cell adhesion molecule 10 25,562,223-25,569,668 No (IBD) ^1,2^

*Rps19* Ribosomal protein S19 25,669,390-25,674,825 No Intracellular protein and IBD^2^

*Arhgef1* Rho guanine nucleotide exchange factor 1 25,687,931-25,711,611 No Intracellular protein

*Rabac1* Rab acceptor 1 (prenylated) 25,754,769-25,757,747 No Membrane bound protein

*Cnfn* Cornifelin 26,152,635-26,154,743) No Envelope protein and IBD^2^

*Cyp2a12* Cytochrome P450, 2a12 27,814,121-27,821,851 No Region IBD^2^

*Cyp2f2* Cytochrome P450, 2f2 27,904,928-27,918,679 No Region IBD^2^

*Pld3* Phospholipase D family, member 3 28,317,023-28,338,186 No Region IBD^2^

*Dll3* Delta-like 3 (*Drosophila*) 29078572-29087257 No Membrane protein and IBD^2^

*Lrfn1* Leucine rich repeat and fibronectin type III

domain containing 1 29,237,257-29,252,567 No Region IBD^2^

*Psenen* Presenilin enhancer 2 homolog (C. elegans) 31,346,887-31,348,203 No Membrane protein processing

*Zbtb32* Zinc finger and BTB domain containing 32 31,374,700-31,383,928 No Nuclear protein

*Upk1a* Uroplakin 1A 31,388,111-31,397,753 No Membrane protein

*Cox6b1* Cytochrome c oxidase, subunit 6b polypeptide 1 31,401,993-31,411,170 No Mitochondrial protein

CD22 antigen 31650423-31665361

*Tmem147* Transmembrane protein 147 31,512,720-31,514,553 No Membrane protein

*Cd22* CD22 antigen 31,650,423-31,665,361 No B-cell receptor

*Hamp* Hepcidin antimicrobial peptide 31,727,390-31,729,036 No Maintains iron homeostasis

*Abpa2* Androgen-binding protein eta (aka ABPA2) 32,075,539-32,076,835 No Microarray error^4^

*Abpbg26* Androgen-binding protein beta-gamma 26 34,728,016-34,730,004 Yes Secreted in large quantities

*Abpbg27* Androgen-binding protein beta-gamma 27 34,796,951-34,798,961 Yes Secreted in large quantities

*Abpa27*  Androgen-binding protein alpha 27 34,806,586-34,807,900 Yes Secreted in large quantities

*Uba2* Ubiquitin-like modifier activating enzyme 2 34,925,708-34,954,618 No Intracellular protein

^1^Peptide(s) found in the MUDPIT analyses under very relaxed criteria: only one peptide and 95% identity (Karn and Laukaitis 2011)

^2^Determined with analysis by the Mouse Phylogeny Viewer to be identical by descent (IBD)

^3^Checked by sequencing – sequences the same or differ by only one or two conservative substitutions

^4^Expression studies with specific PCR primers show that Abpa2 is not expressed in salivary glands but rather in lacrimal gland (Laukait*is et a*l. 2005). ABPA2 has a Leu/Pro substitution at site 91 between C3H and DBA.
