# Supplementary material for: Congenic Strain Analysis Reveals Genes That Are Rapidly Evolving Components of a Prezygotic Isolation Mechanism Mediating Incipient Reinforcement
Source: PLoS One. 2012 Apr 25;7(4):e35898. doi: 10.1371/journal.pone.0035898 (PMC3338474; doi:10.1371/journal.pone.0035898)
Supplement: File S1 — Retained and eliminated proteins (represented by ESTs and cDNAs). (DOC) [file pone.0035898.s005.doc]

File S1. Retained and eliminated proteins (represented by ESTs and cDNAs)

Retained proteins

*Abpa27* Androgen binding protein alpha27 (chr7:34806586-34807900; Accession number IPI127933)

Highly expressed in mouse salivary proteomes [1]; transcript found with specific primers in all three major salivary glands (parotid, submandibular and sublingual; [2]. Likely misidentified in the EST study as Abp eta (Abph; chr7:32075538-32076835; [3]).

*Abpa26* Androgen binding protein beta-gamma26 (chr7: 34728016-34730004; Accession number IPI170392)

Highly expressed in mouse salivary proteomes [1]; transcript found with specific primers in submandibular and sublingual glands but not in parotid [2].

*Abpa27* Androgen binding protein beta-gamma27 (chr7: 34796951-34798961; Accession number IPI153363)

Highly expressed in mouse salivary proteomes [1]; transcript found with specific primers in all three major salivary glands (parotid, submandibular and sublingual; [2].

Eliminated proteins

*Calm3* Calmodulin (chr7:17500728-17509463; Accession number IPI00761696)

Calmodulin mediates processes such as inflammation, metabolism, apoptosis, smooth muscle contraction, intracellular movement, short-term and long-term memory, nerve growth and the immune response. Calmodulin is an intracellular protein expressed in many cell types and can have different subcellular locations, including the cytoplasm, within organelles, or associated with the plasma or organelle membranes. The Perlegen Mouse Ancestry Browser suggests that the C3H/HeJ and DBA/2J strains may have acquired their *Calm3* genes from different subspecies but the intracellular expression of this gene suggests that it is not secreted into saliva. The Sanger Mouse Genomes Project data shows them to be identical at all amino acid sites in t the C3H and DBA mouse strains.

*Ceacam14* Carcinoembryonic antigen-related cell adhesion molecule B1 (chr7:18397982-18401012; Accession number IPI00132346)

This group of proteins includes important tumor markers for colorectal and some other carcinomas. These proteins are members of the carcinoembryonic antigen (CEA) gene family, which belongs to the immunoglobulin superfamily. The Perlegen Mouse Ancestry Browser and the Mouse Phylogeny Viewer both suggest that the C3H/HeJ and DBA/2J strains acquired their *Ceacam14* genes from different subspecies. Moreover, the GNF Expression Atlas 2 data from GNF1M mouse chip shows no *Ceacam14* expression in salivary glands contrary to the weak U74a expression and that reported by [3]. Even under the most relaxed conditions, there is no evidence for peptides of the CEACAM14 protein in the mouse saliva proteomes [1] in spite of the relatively high transcript levels reported by [3]. These are cell membrane associated proteins and are unlikely to be secreted into saliva.

*Pglyrp1* Peptidoglycan recognition protein 1 (chr7:19470039-19475787; Accession number IPI00132111)

Peptidoglycan recognition protein-1 (PGLYRP-1) is part of the innate immune system and binds to peptidoglycan, a component of bacterial cell walls that has been found in human atherosclerotic lesions. The Perlegen Mouse Ancestry Browser is not helpful because it shows a large gap spanning from 19.25 Mb to just over 23 Mb in the DBA/2J strain. The Sanger Mouse Genomes Project data show them to be identical at all amino acid sites in the the C3H and DBA mouse strains.

*Rsph6a* Radial spoke head 6 homolog (Chlamydomonas) (chr7:19640039-19659796; Accession number IPI00408167)

The protein encoded by this gene belongs to the flagellar radial spoke RSP4/6 family and is similar to a sea urchin radial spoke head protein. Radial spoke protein complexes form part of the axoneme of eukaryotic flagella and are located between the axoneme's outer ring of doublet microtubules and central pair of microtubules. In Chlamydomonas, radial spoke proteins are thought to regulate the activity of dynein and the symmetry of flagellar bending patterns. Radial spoke proteins are part of the flagellar machinery of the cell and are not expected to be secreted in exocrine fluids such as saliva.

*Snrpd2* Small nuclear ribonucleoprotein D2 (chr7:19735071-19738084; Accession number IPI00119220)

The protein encoded by this gene belongs to the small nuclear ribonucleoprotein core protein family. Sm proteins are components of the U1, U2, U4/U6 and U5 small ribonucleoproteins (snRNPs). As many as 10 or more proteins may be assembled into these complexes. Some of the Sm proteins are shared between snRNPs while others are specific to particular snRNPs. Among the core ribonucleo-proteins are 3 distinct subunits designated D1, D2, and D3. These are intracellular proteins required for pre-mRNA splicing and small nuclear ribonucleoprotein biogenesis and, as such, they are not expected to be secreted in exocrine fluids such as saliva.

*Ckm* Creatinine kinase, muscle (chr7:19996443-20006932; Accession number IPI00127596)

Creatine (sometimes called creatinine kinase) an enzyme expressed by various tissues and cell types, as well as in serum. CK catalyses the conversion of [creatine](http://en.wikipedia.org/wiki/Creatine) and consumes  ATP to create [phosphocreatine](http://en.wikipedia.org/wiki/Phosphocreatine) (PCr) and  ADP.  In heart, in addition to the MM-CK homodimer also the heterodimer consisting of one muscle (MM-CK) and one brain-type (MB-CK) subunit is expressed. This latter is an important serum marker for myocardial infarction, for it is released from damaged myocardial cells into the blood where it can be detected by clinical chemistry.  There is no reason to believe that this enzyme is secreted into saliva in any quantity and it does not appear in the mouse saliva proteomes [1]. The Perlegen Mouse Ancestry Browser shows a large gap spanning from 19.25 Mb to just over 23 Mb in the DBA/2J strain.

*Apoe* Apolipoprotein E (chr7:20281595-20284515; Accession number IPI00323571)

Apolipoprotein E is a class of [apolipoprotein](http://en.wikipedia.org/wiki/Apolipoprotein) found in the [chylomicron](http://en.wikipedia.org/wiki/Chylomicron) and intermediate-density **lipoproteins (IDLs) that binds to a specific receptor on liver cells and peripheral cells. It is essential for** the normal catabolism of triglyceride-rich lipoprotein constituents. There is no reason to believe that apolipoprotein E is secreted into saliva in any quantity and the Sanger Mouse Genomes Project data shows their amino acid sequences to be identical with the exception of a Glu to Asp difference at position 163 in the C3H and DBA mouse strains. This is a conservative amino acid substitution and is unlikely to create a substantial difference in function nor would it be expected to significantly influence mate choice of mice in preference tests. In addition, apolipoportein E does not appear in the mouse saliva proteomes [1]. The Perlegen Mouse Ancestry Browser is not helpful because it shows a large gap spanning from 19.25 Mb to just over 23 Mb in the DBA/2J strain.

*Ceacam10* Carcinoembryonic antigen-related cell adhesion molecule 10 (chr7:25562223-25569668; Accession number IPI00408931)

### This group of proteins includes **important tumor markers for colorectal and some other carcinomas. These proteins are members of the carcinoembryonic antigen (CEA) gene family, which belongs to the immunoglobulin superfamily.** The Perlegen Mouse Ancestry Browser and the Mouse Phylogeny Viewer both suggest that the C3H/HeJ and DBA/2J strains are identical by descent in the *Ceacam10* region, having acquired their *Ceacam10* genes from *M. m. domesticus*. Even under the most relaxed conditions, there is no evidence for peptides of the CEACAM10 protein **in the mouse saliva proteomes [1] in spite of the relatively high transcript levels reported by [3]. These are cell membrane associated proteins and are unlikely to be secreted into saliva.**

*Rps19* Ribosomal protein S19 (chr7:25669390-25674825; Accession number IPI00113241)

Ribosomal proteins are part of the cellular machinery involved in translation of mRNAs into protein and are not secreted in exocrine fluids such as saliva. The Perlegen Mouse Ancestry Browser suggests that the C3H/HeJ and DBA/2J strains both acquired the *Mus musculus domesticus* genome background in this region of their genomes and thus should not differ in this protein.

*Arhgef1* Rho guanine nucleotide exchange factor (GEF) 1 (chr7:25687931-25711611; Accession number IPI00127009)

Rho GTPases have a critical role in numerous cellular processes that are initiated by extracellular stimuli that work through G protein coupled receptors. The encoded protein may form a complex with G proteins and stimulate Rho-dependent signals. These factors are intracellular proteins and are not secreted in exocrine fluids such as saliva.

*Rabac1* Rab acceptor 1 (prenylated) (chr7:25754769-25757747; Accession number IPI00129399)

Prenylated proteins such as rab acceptor 1 are membrane bound proteins and are not secreted in exocrine fluids such as saliva.

*Cnfn* Cornifelin (chr7:26152635-26154743; Accession number IPI00669433)

Cornifelin in mice has been shown to be directly or indirectly cross-linked to at least two other cornified envelope proteins, loricrin and involucrin, in vivo. The Sanger Mouse Genomes Project data show the amino acid sequences to be identical in the C3H and DBA mouse strains.

*Cyp2a12* and *Cyp2f2* Cytochrome P450, 2a12 (chr7:27814121-27821851) and Cytochrome P450, 2F2 (chr7:27904928-27918679; Accession numbers IPI00308919 and IPI00308328, respectively)

Responding to the question: Are cytochrome P450’s ever secreted into saliva, Xinxin Ding responded (personal communication): “There is no solid study on secretion of CYP; however, (some) people detected P450 in the mucocilliary complex of the olfactory mucosa by immunohistochemistry and proposed that it may be "secreted." It is always possible to have epithelial shedding into secretions, in which case the detected protein will unlikely be active.” The Perlegen Mouse Ancestry Browser and the Mouse Phylogeny Viewer both suggest that the C3H/HeJ and DBA/2J strains are identical by descent in the *Cyp* region that includes these genes, having acquired their cytochrome P450 2a12 and 2F2 genes from *M. m. domesticus*.

*Pld3* Phospholipase D family, member 3 (chr7:28317023-28338186; Accession number IPI00130624)

Phospholipase D (PLD) catalyzes conversion of phosphatidylcholine into choline and phosphatidic acid, leading to a variety of intracellular signal transduction events. The Sanger Mouse Genomes Project data show the amino acid sequences to be identical at all sites in the C3H and DBA mouse strains.

*Dll3* Delta-like 3 (Drosophila) (chr7:29078572-29087257; Accession number IPI00131718)

This gene encodes a member of the delta protein ligand family. This family functions as Notch ligands that are characterized by a DSL domain, EGF repeats, and a transmembrane domain. Mutations in this gene cause autosomal recessive spondylocostal dysostosis 1. The Perlegen Mouse Ancestry Browser and the Mouse Phylogeny Viewer both suggest that the C3H/HeJ and DBA/2J strains are identical by descent in the *Dll3* region, having acquired their *Dll3* genes from *M. m. domesticus*.

*Lrfn1* Leucine rich repeat and fibronectin type III domain containing 1 (chr7:29237257-29252567; Accession number IPI00468602)

The Perlegen Mouse Ancestry Browser and the Mouse Phylogeny Viewer both suggest that the C3H/HeJ and DBA/2J strains are identical by descent in the *Lrfn1* region, having acquired their *Lrfn1* genes from *M. m. domesticus*.

*Psenen* Presenilin enhancer 2 homolog (*C. elegans*) (chr7:31346887-31348203; Accession number IPI00132969)

Presenilins, which are components of the gamma-secretase protein complex, are required for intramembranous processing of some type I transmembrane proteins, such as the Notch proteins and the beta-amyloid precursor protein. Signaling by Notch receptors mediates a wide range of developmental cell fates. These proteins will be membrane bound/associated and are expected to be secreted in exocrine fluids such as saliva.

*Zbtb32* Zinc finger and BTB domain containing 32 (chr7:31374700-31383928; Accession number IPI00122873)

This protein is located in the nucleus, is involved in cell cycle progression; it is not expected to be secreted in exocrine fluids such as saliva.

.

*Upk1a* Uroplakin 1A (chr7:31388111-31397753; Accession number IPI00133059)

Uroplakin 1A is a component of the asymmetric unit membrane (AUM); a highly specialized bio-membrane elaborated by terminally differentiated urothelial cells. It may play an important role in normal bladder epithelial physiology, possibly in regulating membrane permeability of superficial umbrella cells or in stabilizing the apical membrane through AUM/cytoskeletal interactions. As a membrane bound/associated protein, it is not expected to be secreted in exocrine fluids such as saliva.

*Cox6b1* Cytochrome c oxidase, subunit VIb polypeptide 1 (chr7:31401993-31411170; Accession number IPI00225390)

This is a mitochondrial protein and is found in the electron transport chain, a group of membrane-bound proteins. As such, it is not expected to be secreted in exocrine fluids such as saliva.

*Tmem147* Transmembrane protein 147 (chr7:31512720-31514553; Accession number IPI00318696)

Transmembrane protein 147 is located in the [endoplasmic reticulum membrane](http://www.uniprot.org/locations/SL-0097). As a membrane bound/associated protein, it is not expected to be secreted in exocrine fluids such as saliva.

*Cd22* CD22 antigen (chr7:31,650,423-31,665,361; Accession number IPI00117413)

CD22 is a B-cell receptor and is not expected to be secreted in exocrine fluids such as saliva. Homozygous null mice have reduced mature B cell numbers with altered proliferation kinetics and reduced antibody production to T cell independent antigens.

*Hamp* Hepcidin antimicrobial peptide (chr7:31727390-31729036; Accession number IPI00110291)

Hepcidin antimicrobial peptide appears to act as a signaling molecule involved in the maintenance of iron homeostasis. It may be required in conjunction with HFE to regulate both intestinal iron absorption and iron storage in macrophages. It may also have antimicrobial activity. This peptide or parts thereof are not represented among those in the mouse saliva proteomes, Even under the most relaxed conditions [1], in spite of the relatively high transcript levels reported by [3].

*Abpah* Androgen-binding protein eta (aka ABPA2) (chr7:32075539-32076835; Accession number N/A –provided by the author for Scaffold searching)

ABPA2 is encoded by *Abpa2*, a gene on the left flank of the 3 Mb Abp gene region. In an exhaustive transcript analysis of all three salivary glands, using specific primers for *Abpa2* and many other *Abpa* and *Abpbg* transcripts, no expression of *Abpa2* was found in any of the three salivary glands although significant expression was found in lacrimal glands [2], as had been reported by others. Moreover, ABPA2 protein was not found in the saliva proteomes of either male or female C57BL/6 mice in a recent saliva proteomics study [1]. It is likely that the *Abpa2*

transcript reported as the androgen-binding protein eta identification in BALB/c submandibular and sublingual [3] is a misidentification due to “androgen-binding protein eta” probes having significant identity with *Abpa27* transcripts (known to be translated and contribute protein to the saliva proteomes). Furthermore, we checked the C3H and DBA ABPA2 proteins in the Sanger Mouse Genomes Project data and found them to be identical with the exception of a Leu/Pro substitution at residue 91. Alone, this amino acid substitution is unlikely to create a substantial difference in function nor would it be expected to significantly influence mate choice of mice in preference tests.

*Uba2* Ubiquitin-like modifier activating enzyme 2 (chr7:34925708-34954618; Accession number IPI00130173)

The heterodimer acts as a E1 ligase for SUMO1, SUMO2, SUMO3, and probably SUMO4. It mediates ATP-dependent activation of SUMO proteins followed by formation of a thioester bond between a SUMO protein and a conserved active site cysteine residue on UBA2/SAE2Ubiquitin marks proteins for degradation within the cell and the enzymes catalyzing this are not expected to be secreted in exocrine fluids such as saliva.

References:

1. Karn RC, Laukaitis CM (2011) Positive selection shaped the convergent evolution of independently expanded kallikrein subfamilies expressed in mouse and rat saliva proteomes. PLoS ONE 6: e20979.

2. Laukaitis CM, Dlouhy SR, Emes RD, Ponting CP, Karn RC (2005) Diverse spatial, temporal, and sexual expression of recently duplicated androgen-binding protein genes in Mus musculus. BMC Evol Biol 5: 40.

3. Treister NS, Richards SM, Lombardi MJ, Rowley P, Jensen RV, et al. (2005) Sex-related differences in gene expression in salivary glands of BALB/c mice. Journal of Dental Research 84: 160-165.
