# Supplementary figures and images for: Congenic Strain Analysis Reveals Genes That Are Rapidly Evolving Components of a Prezygotic Isolation Mechanism Mediating Incipient Reinforcement
Source: PLoS One. 2012 Apr 25;7(4):e35898. doi: 10.1371/journal.pone.0035898 (PMC3338474; doi:10.1371/journal.pone.0035898)

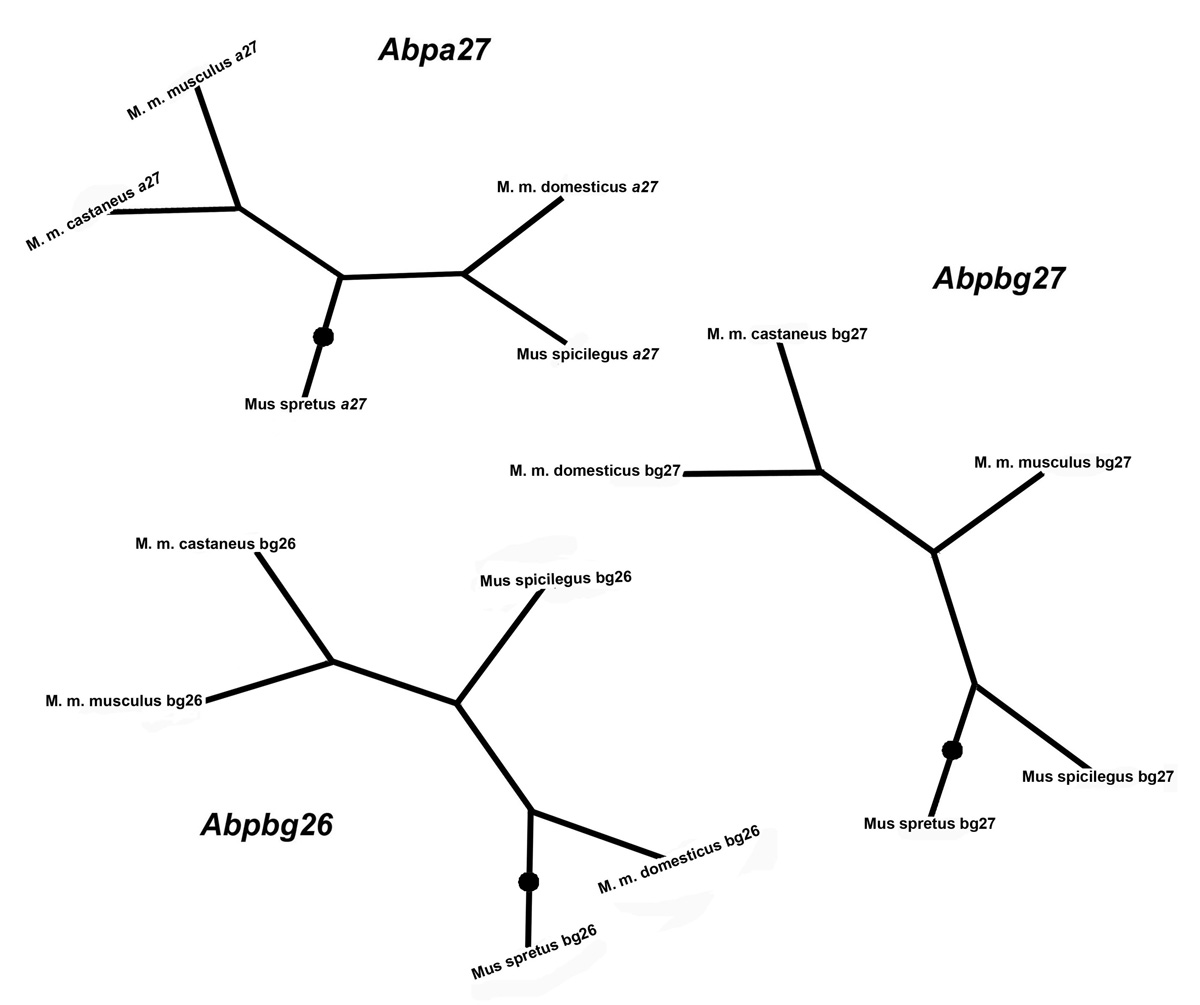

Supplement: File S4 — Unrooted trees for Abpa27 , Abpbg26 and Abpbg27 . Trees were made as in Fig. 3 but were not rooted. Rather, the suggested root is shown by a black dot on the branch to the Mus spretus ortholog. (TIF) [file pone.0035898.s008.tif]
