# Supplementary material for: Congenic Strain Analysis Reveals Genes That Are Rapidly Evolving Components of a Prezygotic Isolation Mechanism Mediating Incipient Reinforcement
Source: PLoS One. 2012 Apr 25;7(4):e35898. doi: 10.1371/journal.pone.0035898 (PMC3338474; doi:10.1371/journal.pone.0035898)
Supplement: File S5 — Details of Materials and Methods. (DOC) [file pone.0035898.s009.doc]

File S5. Details of Materials and Methods

All animal manipulation was performed humanely and under appropriate Animal Welfare Guidelines under University of Arizona IACUC protocol 08-138.

*Materials*

Genomic DNAs from the following wild-derived inbred strains were obtained from Jackson Laboratory: *Mus musculus domesticus* strain WSB/EiJ, ; *M. m. musculus* strain PWK/PhJ; *M. m. castaneus* strain CAST/EiJ; *M. spicilegus* strain PANCEVO/EiJ; *M. spretus* strain SPRET/EiJ; and *M. caroli* strain CAROLI/EiJ. PCR and DNA sequencing primers were obtained from Bioneer. Primer sequences and conditions are available from the authors upon request.

*Molecular methods*

DNA isolation from b-congenic spleens or tail tips was adapted from [1]. Tissue was lysed in lysis buffer with Proteinase K at 55 ^o^C overnight and centrifuged. DNA was precipitated from the supernatant by the addition of isopropanol, incubation at room temperature and centrifugation. The pellet was washed with cold 70% Ethanol and centrifuged. The DNA was air dried 30 min and then re-suspended in 50-100 ul of water.

Polymerase chain reaction (PCR) was run as previously described [2] and the products were evaluated on 1% agarose gels, diluted 1:4 with distilled water, and sequenced either by the UAGC facility at the University of Arizona or by MCLAB (<http://www.mclab.com/)>. Gene sequences obtained from other species were checked against their known mouse genome coordinates (cds) with the BLAT tool on the UCSC genome browser (<http://genome.ucsc.edu/>; [3,4]).

*Mouse salivary gland expression data sets*

Salivary gland protein expression data sets were obtained by searching “mouse salivary gland” on the NCBI Gene Expression Omnibus website ([www.ncbi.nlm.nih.gov/geo/](http://www.ncbi.nlm.nih.gov/geo/)). The most complete set we encountered contained ESTs found in submandibular (aka submaxillary) glands (3 replicates of each sex), sublingual glands (2 replicates of each sex) and parotid glands (2 replicates of each sex) of BALB/c mice [5]. The data sets were acquired from the GEO Data Set archives on pubmed under project record number [GDS1009](http://www.ncbi.nlm.nih.gov/sites/GDSbrowser?acc=GDS1009)  Project Title: *Male and female salivary gland comparison.* The platform [GSE1503](http://www.ncbi.nlm.nih.gov/geo/query/acc.cgi?acc=GSE1503) consisting of Amersham CodeLink UniSet Mouse I Bioarray was downloaded and cross referenced with expression-profiling data of 9-week-old male and female BALB/c parotid, submandibular, and sublingual gland data sets. The submandibular set consisted of 3 female ([GSM25992](http://www.ncbi.nlm.nih.gov/geo/query/acc.cgi?acc=GSM25992), [GSM25993](http://www.ncbi.nlm.nih.gov/geo/query/acc.cgi?acc=GSM25993), [GSM25994](http://www.ncbi.nlm.nih.gov/geo/query/acc.cgi?acc=GSM25994)) and 3 male data sets ([GSM25995](http://www.ncbi.nlm.nih.gov/geo/query/acc.cgi?acc=GSM25995), [GSM25996](http://www.ncbi.nlm.nih.gov/geo/query/acc.cgi?acc=GSM25996), [GSM25997](http://www.ncbi.nlm.nih.gov/geo/query/acc.cgi?acc=GSM25997)) each labeled A, B, C accordingly. Sublingual and parotid data sets only contained 2 sets for each mouse A and B (sublingual: Female [GSM25998](http://www.ncbi.nlm.nih.gov/geo/query/acc.cgi?acc=GSM25998), [GSM25999](http://www.ncbi.nlm.nih.gov/geo/query/acc.cgi?acc=GSM25999), male [GSM26000](http://www.ncbi.nlm.nih.gov/geo/query/acc.cgi?acc=GSM26000), [GSM26001](http://www.ncbi.nlm.nih.gov/geo/query/acc.cgi?acc=GSM26001) and parotid: Female [GSM26002](http://www.ncbi.nlm.nih.gov/geo/query/acc.cgi?acc=GSM26002), [GSM26003](http://www.ncbi.nlm.nih.gov/geo/query/acc.cgi?acc=GSM26003), male [GSM26004](http://www.ncbi.nlm.nih.gov/geo/query/acc.cgi?acc=GSM26004), [GSM26005](http://www.ncbi.nlm.nih.gov/geo/query/acc.cgi?acc=GSM26005)). Data was downloaded and cross-referenced with Amersham Codelink UniSet Mouse 1 Bioarray, and protein information for each probe target was gathered by searching probe accession numbers on MGI (<http://www.informatics.jax.org/>), the international database resource for the laboratory mouse. The data in the region of interest was also sorted on the sample signal intensity from the array, which ranged from 0 to 250-400 in the six data sets, and only those with values of 10 or above were retained for this study. The lower values each represented less than 4% of the highest value in the set and yet the collection of each discarded group was 90% or more of the signals identified. In this way, we focused on those ESTs most likely to represent gene expression that could have contributed proteins to the mouse saliva. The UCSC Mouse Genome Browser was used to sort and save the chromosome 7 encoded genes, along with their cds and their gene names.

*Data analysis*

DNA sequence traces were edited with Chromas 2.3 (http://www.technelysium.com.au). DNA sequence alignment, coding region assembly, and *in silico* translation were done using the DNAsis Max program 2.0 (Hitachi). We used the Perlegen Mouse SNP Browser (<http://mouse.cs.ucla.edu/perlegen>) and the Mouse Phylogeny Viewer (<http://msub.csbio.unc.edu/>) to assess the subspecies origin of various segments of the proximal end of mouse chromosome 7, as well as in the construction of figures. The UCSC browser [6]; [http://www.genome.ucsc.edu](http://www.genome.ucsc.edu/)) was used to obtain the DNA sequences of genes in the area of interest on mouse chromosome 7. Permission was obtained, with permission, from the Mouse Genomes Project (<http://www.sanger.ac.uk/Projects/M_musculus/>), Sanger Institute, to download whole genome sequences for eight mouse strains of which the C3H/HeJ, CAST/EiJ, DBA/2J, PWK/PhJ, SPRET/EiJ, and WSB/EiJ data were used in this study. Orthology was confirmed by reciprocal Blast searches against the mouse genome [7]. The chromosome 7 sequence for each strain was extracted using the High Performance Computing system from the University of Arizona Biotechnology Computing Facility.

Positive selection was assessed in the program CODEML in the PAML package [8-10]. The three subspecies of *M. musculus* were treated as an unresolved polytomy in the species guide tree. For each gene, three different comparisons of neutral and selection models gave similar results (M1 vs. M2, M7 vs. M8, and M8A vs. M8 [11-13]. Model M1 (neutral) allows two classes of codons, one with *dN/dS* over the interval (0,1) and the other with a *dN/dS* value of one. Model M2 (selection) is similar to M1 except that it allows an additional class of codons with a freely estimated *dN/dS* value. Model M7 (neutral) estimates *dN/dS* with a beta-distribution over the interval (0, 1), whereas model M8 (selection) adds parameters to M7 for an additional class of codons with a freely estimated *dN/dS* value. M8A (neutral) is a special case of M8 that fixes the additional codon class at a *dN/dS* value of one. The three-dimensional structures of mouse *a27*, *bg26* and *bg27* were modeled using the PHYRE2 (version 2.0) threading program (http://www.sbg.bio.ic.ac.uk/phyre2/html/page.cgi?id=index; [14], and the resulting models were visualized using PYMOL (open-source 1.2.8; http://www.pymol.org/). Sites under positive selection were mapped onto the structural models using PYMOL and incorporated into a figure.

*References:*

1. Laird PW, Zijderveld A, Linders K, Rudnicki MA, Jaenisch R, et al. (1991) Simplified mammalian DNA isolation procedure. Nucleic Acids Res 19: 4293.

2. Laukaitis CM, Dlouhy SR, Emes RD, Ponting CP, Karn RC (2005) Diverse spatial, temporal, and sexual expression of recently duplicated androgen-binding protein genes in Mus musculus. BMC Evol Biol 5: 40.

3. Kent WJ (2002) BLAT--the BLAST-like alignment tool. Genome Res 12: 656-664.

4. Rhead B, Karolchik D, Kuhn RM, Hinrichs AS, Zweig AS, et al. (2010) The UCSC Genome Browser database: update 2010. Nucleic Acids Res 38: D613-619.

5. Treister NS, Richards SM, Lombardi MJ, Rowley P, Jensen RV, et al. (2005) Sex-related differences in gene expression in salivary glands of BALB/c mice. Journal of Dental Research 84: 160-165.

6. Karolchik D, Baertsch R, Diekhans M, Furey TS, Hinrichs A, et al. (2003) The UCSC Genome Browser Database. Nucleic Acids Research 31: 51-54.

7. Karn RC, Clark NL, Nguyen ED, Swanson WJ (2008) Adaptive evolution in rodent seminal vesicle secretion proteins. Mol Biol Evol 25: 2301-2310.

8. Nielsen R, Yang Z (1998) Likelihood models for detecting positively selected amino acid sites and applications to the HIV-1 envelope gene. Genetics 148: 929-936.

9. Yang Z (1997) PAML: a program package for phylogenetic analysis by maximum likelihood. Comput Appl Biosci 13: 555-556.

10. Yang Z, Swanson WJ, Vacquier VD (2000) Maximum-likelihood analysis of molecular adaptation in abalone sperm lysin reveals variable selective pressures among lineages and sites. Mol Biol Evol 17: 1446-1455.

11. Bielawski JP, Yang Z (2003) Maximum likelihood methods for detecting adaptive evolution after gene duplication. J Struct Funct Genomics 3: 201-212.

12. Swanson WJ, Nielsen R, Yang Q (2003) Pervasive adaptive evolution in mammalian fertilization proteins. Mol Biol Evol 20: 18-20.

13. Yang ZH, Bielawski JP (2000) Statistical methods for detecting molecular adaptation. Trends in Ecology & Evolution 15: 496-503.

14. Kelley LA, Sternberg MJ (2009) Protein structure prediction on the Web: a case study using the Phyre server. Nat Protoc 4: 363-371.
